# Suppression of Common-Mode Resonance in Multiband Base Station Antennas

**DOI:** 10.3390/s23062905

**Published:** 2023-03-07

**Authors:** Madiha Farasat, Dushmantha Thalakotuna, Yang Yang, Zhonghao Hu, Karu Esselle

**Affiliations:** School of Electrical and Data Engineering, University of Technology, Ultimo, Sydney 2007, Australia

**Keywords:** base station antenna, common-mode resonance, common-mode suppression, radiation pattern distortion, wideband matching, 5G, 4G, gNB

## Abstract

5G demands a significant increment in the number of connected devices. As a result, gNodeBs are constantly pushed to serve more spectrum and smaller sectors. These increased capacity demands are met by using multiband antennas in base stations. One of the key challenges with multiband antennas is the pattern distortions due to the presence of other surrounding antenna element structures. This work provides a novel approach to address the challenge of pattern distortion in the lower frequency band 690–960 MHz due to common-mode (CM) currents in the high- frequency-band antenna element operating in the 1810–2690 MHz band. A common-mode suppression circuit is integrated with the impedance matching network of the high-band antenna element to reduce these common-mode currents. The experimental results verified that the common-mode suppression circuit reduces the common-mode currents at low-band frequencies by moving the common-mode resonance frequency outside the low frequency band, resulting in cleaner low-band patterns meeting pattern specifications.

## 1. Introduction

5G access network marks a significant milestone in the evolution of mobile communication. The 5G access network is designed to handle an increased traffic demand [1]. In mobile communication, antennas play a vital role translating the analog circuit signals to electromagnetic waves to propagate through air [2]. These antennas in the last access element, i.e., gNBs in 5G or NodeBs in 4G, are commonly referred to as base station antennas (BSA) [3]. BSAs have evolved from omni-directional single-band antennas from early generations to sectorized multiband multibeam antennas in 5G [4]. To meet the capacity demands for operators, the base station antenna needs to be multiband as this improves the resource utilization. On top, the size of a multiband antenna shall not vary a lot compared to a legacy single-band antenna. Thus, within the same size constraints, a 5G BSA should support multiple frequency bands such as 617 MHz–960 MHz (Low Band),1695 MHz–2180 MHz (High Band 1), 2490 MHz–2690 MHz (High Band 2), and 3300 MHz–3800 MHz (High Band 3) [5]. 

Common practice is to have separate antenna arrays to cover each of these bands. In doing so, antenna arrays will be interspersed on the same ground plane. The challenges in such designs include pattern distortions [6] due to scattering from nearby elements. The pattern distortions can be of two types. First is the high-band (HB) pattern distortions due to nearby low-band (LB) antenna elements. The second is the LB pattern distortions due to the HB antenna element radiating in its common mode (CM). The first is overcome by introducing choking techniques [7], implementation of metal baffles [8,9] and frequency-selective surfaces [10,11]. However, the second challenge of HB common-mode radiation at LB [12] has not been investigated extensively. 

The LB pattern distortions due to HB CM is observed through the impacts on 3dB beamwidth and cross-polarization levels. The 3dB beamwidth of LB patterns will exhibit a significant widening, and cross-polarization levels can be high due to CM radiation. Often it is attempted to reduce the common-mode (CM) resonance by tuning the dimensions of the HB elements. The common approaches are to include capacitive element in the feed, thus moving the CM resonance to higher frequency or out of band [12]. This approach causes broadening of the azimuth beamwidth at lower frequency for commonly used LB radiators.

In this work, we propose a novel approach to reduce the CM resonance by adding a CM suppression circuit. We introduce a CM suppression circuit to the impedance matching network of the HB radiator to minimize the induced CM currents at LB frequencies. This CM suppression circuit is designed to present a high impedance to the HB matching circuit in order for it to appear as an open circuit. 

## 2. The Effect of Common Mode on Low-Band Patterns

**Figure 1:**
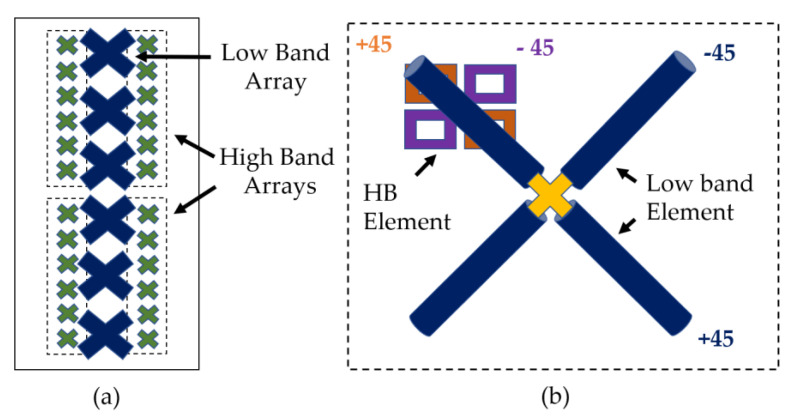
(**a**) Slant dipole configuration used in traditional interspersed scheme for dual-band dual-polarized BSA (**b**) Schematic of the experimental setup with one LB and one HB element.

Typical interspersed HB and LB elements in a modern day multiband BSA are shown in Figure 1a. Due to the use of ±45 polarized elements, the dipoles are oriented in a slant configuration. 

**Figure 2:**
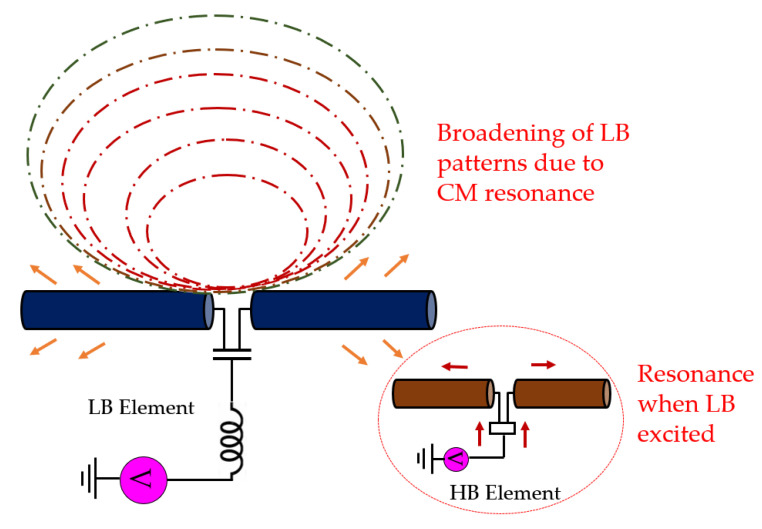
Artistic impression of the common-mode currents induced in nearby High-band (HB) elements impacting the patterns.

**Figure 3:**
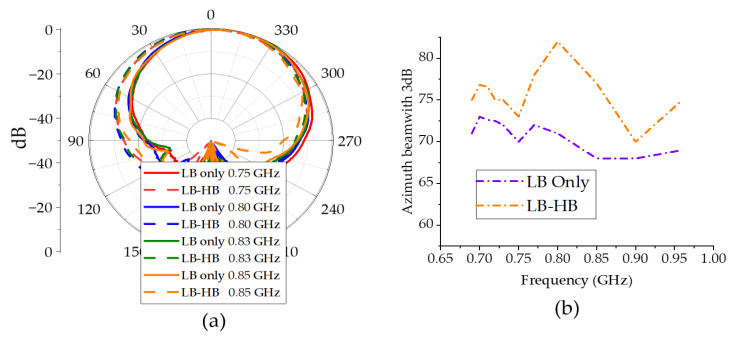
(**a**) Low-band-only (LB-only) and low-band with an HB antenna element (LB-HB) azimuth +45 co-pol patterns. (**b**) Measured 3dB azimuth beamwidth of LB element.

In such an interspersed arrangement, there are two types of resonances that can cause pattern distortions, namely common-mode (CM) resonance and differential mode (DM) resonance. The presence of these resonances is not a problem as long as they occur outside the frequency bands of interest. However, since the total length of the HB feed circuit and the dipoles are approximately a quarter wavelength of LB, when LB radiates it can induce strong common-mode currents on HB elements as shown in Figure 2. Due to these high CM currents, the HB dipole operates as a quarter-wave monopole at LB frequencies. This monopole-like radiation pattern from HB elements at LB frequencies distorts the LB radiation patterns. Here, to clearly present the impact of HB element on performance of the LB element, a parametric study is performed whose results are listed in Table 1. The LB element patterns in the absence of HB element have a half-power beamwidth (HPBW) around 65 + 5°, as shown in Figure 3a. However, with the CM resonance caused by the HB element, the HPBW broadens to 75°–85°, as indicated in Figure 3a,b. 

In order to demonstrate the effect of CM resonance, a simpler dual-band antenna setup is constructed. A schematic representation of this antenna setup is shown in Figure 1b. Only one LB element and one HB element are used from the interspersed array to keep the simulation setup simpler. As demonstrated later, the CM effects are observed even with only one HB element. The LB patterns were measured in the 690–960 MHz band, as it covers the typical BSA LB frequencies. 

## 3. The Working Principle of CM Suppression Circuit

**Figure 4:**
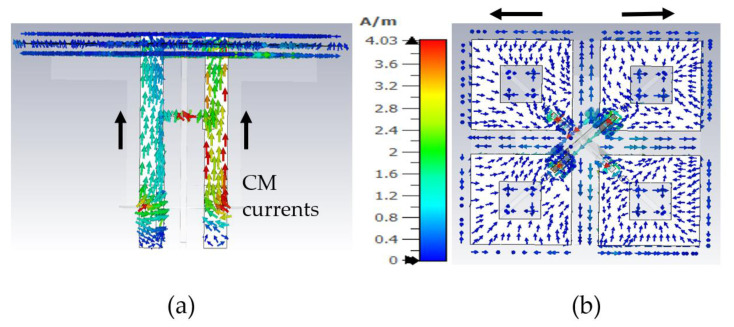
Currents on high-band antenna element at LB frequency 0.69GHz: (**a**) side view; (**b**) top view showing the HB dipole.

A careful observation of the current distribution on the HB dipole shows that the currents on the feed board (stalk) and dipoles travel in the same direction, mimicking the current distribution of a monopole, as shown in Figure 4a,b, when LB patterns are distorted. Only the current distribution at 0.69GHz on an HB antenna is shown in the Figure 4a,b. The currents on HB elements show similar behavior for other frequencies where LB patterns are distorted. An LB antenna element is located near the HB antenna element in this simulation setup and is differentially excited. In order to minimize this CM resonance, the effective resonance length of the HB antenna element at the LB frequencies needs to be altered. At the moment, the length of the HB dipole and the height of the stalk is 35mm, which is λ_glb_/4 at LB frequencies, where λ_glb_/4 is the guided wavelength at low-band mid frequency.

**Figure 5:**
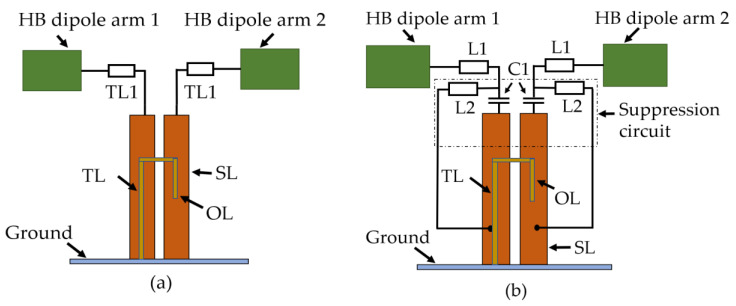
Schematic diagram of (**a**) typical HB antenna feed with the dipoles; (**b**) modified HB antenna feed with the CM suppression circuit. The TL refers to (transmission line); OL (open line); and SL (short line).

A typical HB antenna feed with the dipole is shown in Figure 5a. The feed point of the HB antenna is at the bottom of the TL (transmission line) below the ground plane. The TL and OL (open line) act as an impedance transformer from unbalanced to balanced feed; the balanced SL (short line) and TL1(transmission line 1) are printed at the back of the substrate. Further details on this Balun design can be found in [13]. 

In order to avoid LB currents in the HB stalk, we introduce a common-mode suppression circuit (CMSC) between the dipoles and the balanced feed as shown in Figure 5b. Effectively, the CM suppression circuit should allow all the HB currents to flow as usual while the LB currents are bypassed. The introduction of C1 in the CMSC provides a high series impedance to the LB currents and forces them to flow to ground via the series transmission line L2. Just having a C1 is not sufficient to avoid common-mode currents at LB for this dipole. Therefore, providing a shorting path for LB currents through L2 is necessary. The L2 length is selected such that it is approximately λ_gHB_/4 at HB, where λ_gHB_ is the guided wavelength at HB. Since one end of this L2 is shorted to the ground, it presents an open circuit to HB currents, forcing them to go through C1. The value of the C1 is tuned in CST such that it provides low impedance at HB frequencies and high impedance at LB frequencies. This will ensure that the HB feed circuit operates as a conventional HB feed without a CMSC. 

## 4. Implementation of CM Suppression Circuit on HB

**Figure 6:**
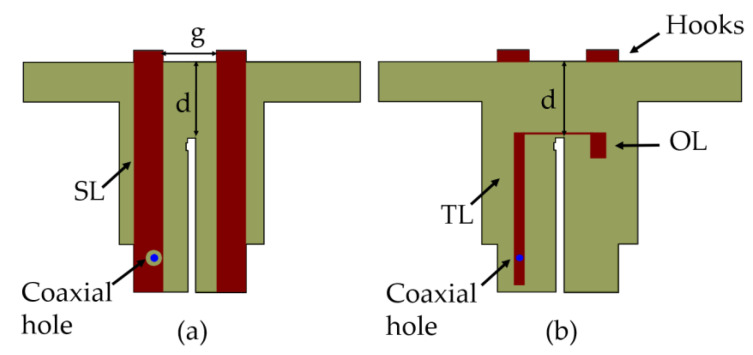
Schematic representation of a typical HB antenna element with impedance matching circuit (**a**) Back view, (**b**) Front view.

**Figure 7:**
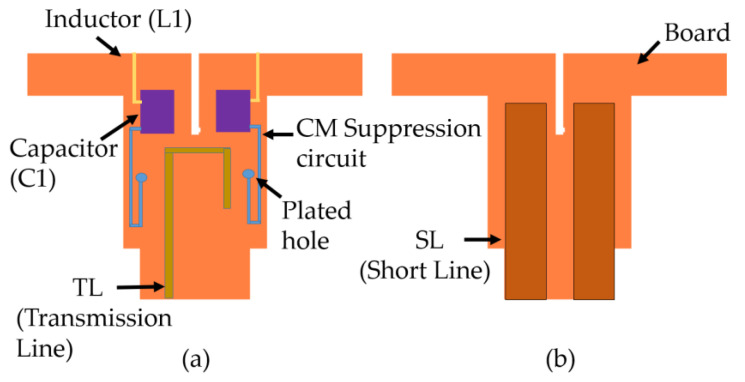
Schematic representation of HB antenna element stalk with CMSC (**a**) Front view, (**b**) Back view.

A conventional HB antenna element feed without the CMSC is shown in Figure 6. Implementing C1 on the feed board can be done either as printed capacitor or an external lumped capacitor while the latter is undesirable due to cost and additional effort for assembly. Implementing the C1 as a parallel plate capacitor using PCB technology is in fact cost-effective and requires no additional effort during the assembly process. The C1 is therefore printed as a parallel plate capacitor as shown in Figure 7. 

**Figure 8:**
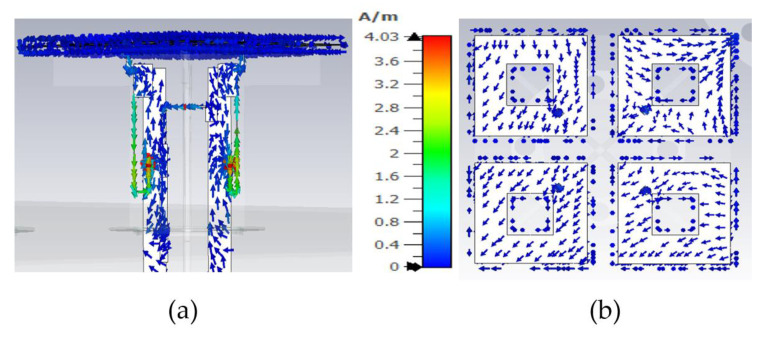
Currents on high-band antenna element with CMSC at LB frequency 0.69 GHz: (**a**) side view; (**b**) top view showing the HB dipole.

The realized capacitance of the C1 is 0.58pF. The capacitance C1 and length of the L2 transmission line were tuned during the simulation to minimize CM LB currents while observing pattern performance during parametric study. The LB CM currents on the HB antenna element are not visible anymore with the CMSC as shown in Figure 8; only differential currents are observed on the HB antenna element, which does not radiate effectively due to mismatch. 

## 5. Experimental Results

**Figure 9:**
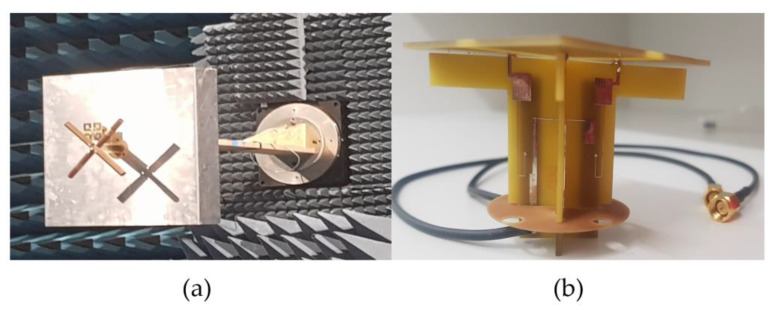
(**a**) The experimental setup consisting of one LB element and one HB element; (**b**) fabricated HB antenna element containing CMSC.

**Figure 10:**
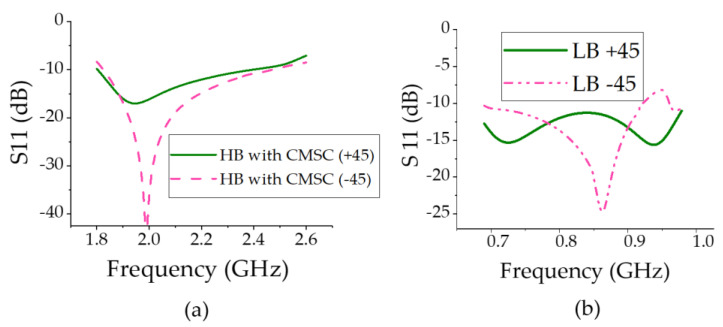
The measured return loss of the (**a**) HB element and (**b**) LB element.

The experimental setup consists of one LB element and one HB element as shown in Figure 9a. Based on simulations, it was found even one HB element near the LB element was sufficient to cause pattern distortions. A conventional slant LB dipole [14] was used as the LB element. The LB dipole impedance matching from 690–960 MHz is based on a feed network design that includes series, shunt resonators, and a quasi-quarter-wavelength transmission line (TL2). A circuit theory model of matching circuit and implementation is proposed in [15]. Both the HB and LB antenna elements achieved S11 < −10 dB across the frequency band of interest as shown in Figure 10. The optimized dimensions of the structure are provided in Table 2.

**Figure 11:**
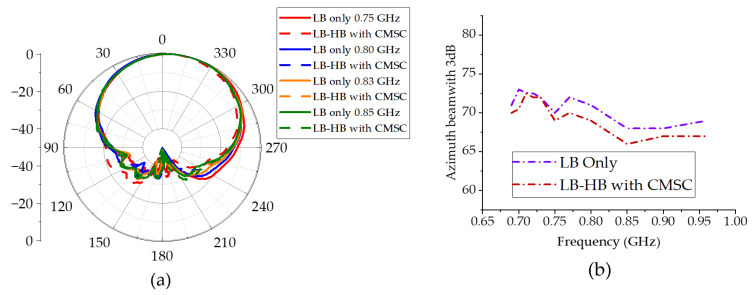
(**a**) Low-band-only (LB only) and low-band with HB antenna element containing CMSC (LB-HB with CMSC) azimuth +45 co-pol patterns. (**b**) Measured 3dB azimuth beamwidth of LB patterns.

**Figure 12:**
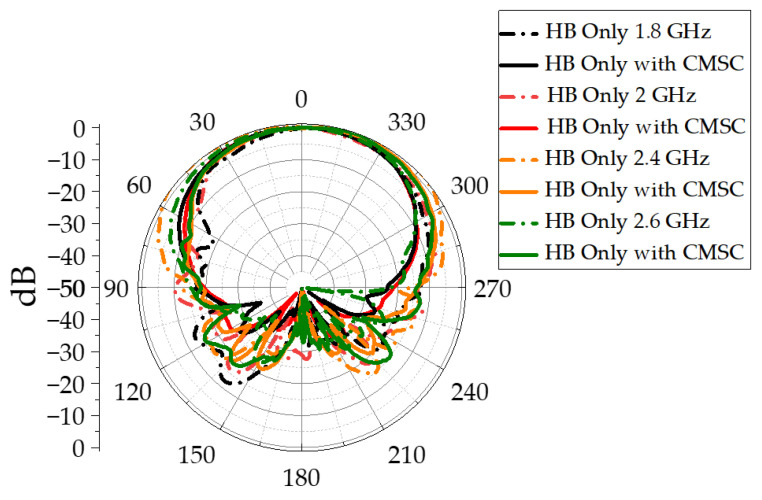
High-band-only antenna element azimuth co-pol patterns.

**Figure 13:**
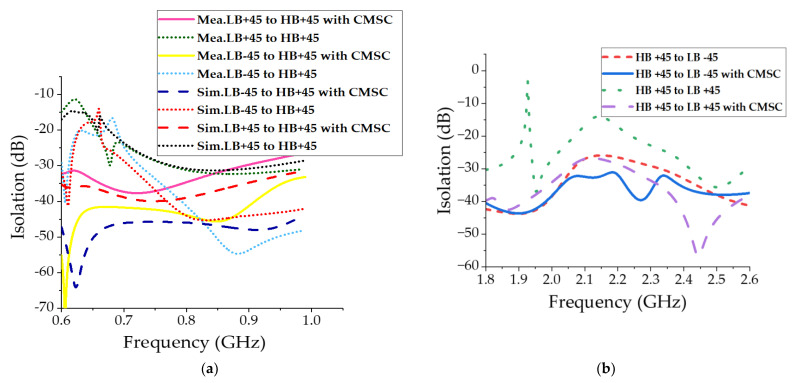
Interband isolation between LB and HB at (**a**) LB frequencies and (**b**) HB frequencies.

Figure 11a shows the measured far field patterns at LB. With the inclusion of CMSC in HB elements, the LB radiation patterns in Figure 11a show a significant improvement compared to patterns in Figure 3a. As indicated in Figure 11b, the 3dB beamwidth is very close to the 3dB beamwidth of the LB element alone, completely removing the broadening effect due to common-mode currents. The HB patterns with and without the CMSC are shown in Figure 12. It is shown that the HB patterns are almost identical and show no impact due to the CMSC. The interband isolation between the LB and HB elements is measured as shown in Figure 13. For clarity, the isolation between HB +45 polarization from the LB polarizations is shown. Without the CMSC, both LB +45 and −45 slant polarizations have high coupling at the bottom of the low-band frequencies. With the CMSC, the coupling is reduced by over 10dB for the same polarization, while it is more than 20dB for opposite polarizations. This significant decoupling also provides an indication of transparency of the HB antenna element to LB frequencies. The simulated values also agree very closely with the measurements from the experimental setup. To address the advantages of the proposed design, a comparison of results with the previous work on mutual coupling suppression techniques is tabulated in Table 3.

## 6. Conclusions

The LB pattern distortions in multiband antennas occur due to common-mode resonance currents induced in HB antenna elements. This causes significant distortions in LB radiation patterns. This is very undesirable for the network performance as it leads to inter-cell interference in adjacent sectors due to coverage overlaps, resulting in degradation of network quality. The common mode is suppressed by introducing a capacitor with a quarter-wavelength short line at LB frequencies to the HB feed network. This suppresses the LB currents at resonance frequencies without significantly changing the HB current distribution at HB frequencies. As a result, CM resonance behavior of the HB dipole is no longer visible, resulting in cleaner patterns at low-band frequencies. The 3 dB beamwidth variation is 65° + 5°. The HB patterns are not affected and the HB element impedance matching below 10 dB can be obtained.

## Figures and Tables

**Table 1:** LB key patterns performance parameters impacted due to HB element.

Frequency (GHz)	Beamwidth (Deg.)	Squint (Deg.)
0.66	82.5	−6
0.67	82.1	−5
0.72	86.4	10
0.75	77.7	16
0.77	75	14
0.85	68.8	5
0.90	66.1	2

**Table 2:** Optimized parameters of the proposed antenna.

Parameters	Values HB (mm)	Description
W-SL	6	Width of SL
L-SL	43	Length of SL
W-TL	1.3	Width of TL
L-TL	15	Length of TL
W-TL1	1	Width of TL1
L-TL1	5.5	Length of TL1
W-TL2	0.2	Width of TL2
L-TL2	20	Length of TL2
W-OL	2.8	Width of OL
L-OL	6	Length of OL
g	11	Gap between SL

**Table 3:** Comparison of recent state-of-the-art works with proposed work.

References	Mutual Coupling Suppression Techniques	Frequency Band (GHz)	Isolation (dB)	HPBW (Measured)
[2]	Passive dipoles + baffles	0.69–0.96 1.7–2.7	>27 >22	72° ± 2° 65° ± 5°
[10]	Frequency-selective surface	0.69–0.96 3.5–4.9	>28 >25	60° 75°
[11]	Frequency-selective surface	2.3–2.7 3.3–3.8	>25 23.5	44°–48° 66°–69°
[12]	Capacitance-loading technique/chokes	0.70–0.96 1.7–2.2	>20	75° + 5° 64° + 5°
[16]	Filtering antenna elements	1.71–1.88 1.9–2.17	>30	65° ± 5°
[17]	Decoupling network	2.3–2.4 2.4–2.483	>25	60° ± 5° 65° ± 5°
[18]	Metal baffles	0.77–0.98 1.65–2.9	<23 17.5	64.5°–57.1° 84.4°–74.1°
[19]	Capacitance-loaded HB element	0.69–0.96 1.7–2.2	>20	75° + 5° 64° + 5°
Proposed Work	Common mode suppression circuit	0.69–0.96 1.8–2.6	>30 >25	65° + 5° 65° ± 5°

## Data Availability

Not applicable.

## References

[1] Osseiran A., Parkvall S., Persson P., Zaidi A., Magnusson S., Balachandran K. (2020). 5G Wireless Access: An Overview.

[2] Huang H., Li X., Liu Y. (2020). A dual-broadband base station antenna with ikebana-like arrangement scheme. Microw. Opt. Technol. Lett..

[3] Farasat M., Thalakotuna D.N., Hu Z., Yang Y. (2021). A review on 5G sub-6 GHz base station antenna design challenges. Electronics.

[4] Beckman C., Lindmark B. The evolution of base station antennas for mobile communications. Proceedings of the 2007 International Conference on Electromagnetics in Advanced Applications.

[5] Thalakotuna D.N., Karmokar D.K., Hu Z., Esselle K.P., Matekovits L. Improving Cross-Band Isolation in MultiBand Antennas. Proceedings of the 2021 International Conference on Electromagnetics in Advanced Applications (ICEAA).

[6] Sun H.H., Ding C., Zhu H., Jones B., Guo Y.J. (2019). Suppression of Cross-Band Scattering in Multiband Antenna Arrays. IEEE Trans. Antennas Propag..

[7] Sun H.-H., Zhu H., Ding C., Jones B., Guo Y.J. (2020). Scattering suppression in a 4G and 5G base station antenna array using spiral chokes. IEEE Antennas Wirel. Propag. Lett..

[8] Huang H., Liu Y., Gong S. (2017). A Dual-Broadband, Dual-Polarized Base Station Antenna for 2G/3G/4G Applications. IEEE Antennas Wirel. Propag. Lett..

[9] He Y., Pan Z., Cheng X., He Y., Qiao J., Tentzeris M.M. (2015). A Novel Dual-Band, Dual-Polarized, Miniaturized and Low-Profile Base Station Antenna. IEEE Trans. Antennas Propag..

[10] Zhu Y., Chen Y., Yang S. (2019). Decoupling and Low-Profile Design of Dual-Band Dual-Polarized Base Station Antennas Using Frequency-Selective Surface. IEEE Trans. Antennas Propag..

[11] Yang S.J., Zhang X.Y. (2021). Frequency Selective Surface-Based Dual-Band Dual-Polarized High-Gain Antenna. IEEE Trans. Antennas Propag..

[12] Sun H.-H., Jones B., Guo Y.J., Lee Y.H. Dual-Band Base Station Antenna Array with Suppressed Cross-Band Mutual Scattering. Proceedings of the 2021 IEEE International Symposium on Antennas and Propagation and USNC-URSI Radio Science Meeting (APS/URSI).

[13] Ding C., Sun H., Ziolkowski R.W., Guo Y.J. (2017). Simplified Tightly-Coupled Cross-Dipole Arrangement for Base Station Applications. IEEE Access.

[14] Ding C., Jones B., Guo Y.J., Qin P.Y. (2017). Wideband Matching of Full-Wavelength Dipole with Reflector for Base Station. IEEE Trans. Antennas Propag..

[15] Farasat M., Thalakotuna D., Hu Z., Yang Y. (2022). A Simple and Effective Approach for Scattering Suppression in Multiband Base Station Antennas. Electronics.

[16] Zhang Y., Zhang X.Y., Ye L., Pan Y. (2016). Dual-Band Base Station Array Using Filtering Antenna Elements for Mutual Coupling Suppression. IEEE Trans. Antennas Propag..

[17] Zhao L., Qian K.W., Wu K.L. (2014). A Cascaded Coupled Resonator Decoupling Network for Mitigating Interference Between Two Radios in Adjacent Frequency Bands. IEEE Trans. Microw. Theory Tech..

[18] Huang H., Liu Y., Gong S. (2016). A Novel Dual-Broadband and Dual-Polarized Antenna for 2G/3G/LTE Base Stations. IEEE Trans. Antennas Propag..

[19] Sun H.-H., Jones B., Guo Y.J., Lee Y.H. (2020). Suppression of cross-band scattering in interleaved dual-band cellular base-station antenna arrays. IEEE Access.

